# Supplementary material for: An Algorithm Combining Patient Performance Status, Second Hit Analysis, PROVEAN and Dann Prediction Tools Could Foretell Sensitization to PARP Inhibitors in Digestive, Skin, Ovarian and Breast Cancers
Source: Cancers (Basel). 2021 Jun 22;13(13):3113. doi: 10.3390/cancers13133113 (PMC8268870; doi:10.3390/cancers13133113)
Supplement: Supplementary file 1 [file cancers-13-03113-s001.zip › cancers-1224008-supplementary.pdf]

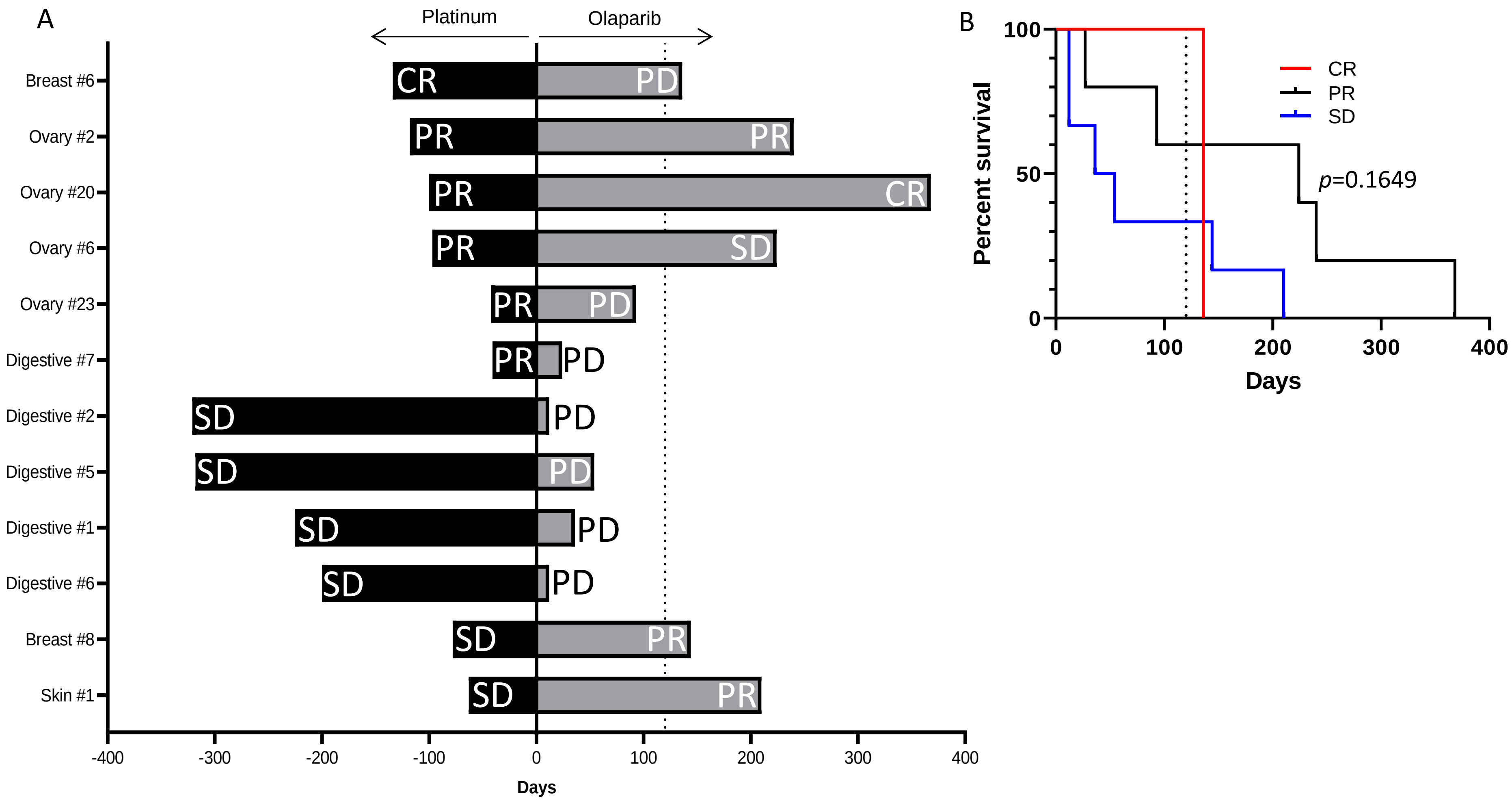
A
Platinum
Olaparib
Breast #6
Ovary #2
Ovary #20
Ovary #6
Ovary #23
Digestive #7
Digestive #2
Digestive #5
Digestive #1
Digestive #6
Breast #8
Skin #1
CR
PD
PR
PR
PR
CR
PR
SD
PR
PD
PR
PD
SD
PD
SD
PD
SD
PD
SD
PD
SD
PR
SD
PR
-400
-300
-200
-100
0
100
200
300
400
Days
B
100
50
0
Percent survival
CR
PR
SD
p=0.1649
0
100
200
300
400
Days

Supplementary Figure S1: Response of VUS to platinum. A. Duration and response (PD: Progressive Disease, SD: Stable Disease, PR: Partial Response, CR: Complete Response) to platinum treatment and subsequent olaparib treatment for VUS. The dashed line corresponds to 120 days. **B.** PFS curve of patients under olaparib treatment depending on their response to platinum based treatment.

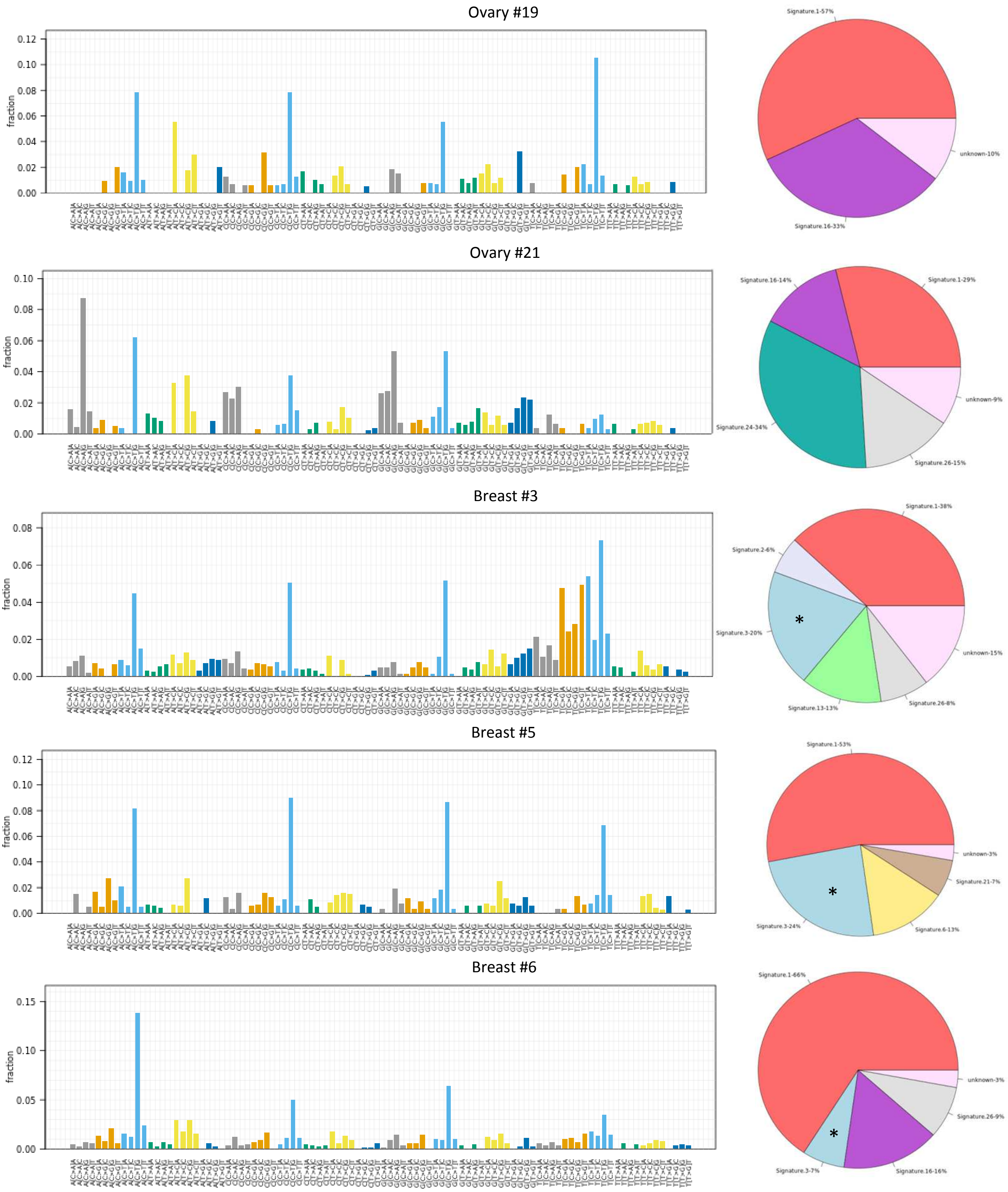
Ovary #19
fraction
Signature.1-57%
unknown-10%
Signature.16-33%
Ovary #21
fraction
Signature.16-14%
Signature.1-29%
unknown-9%
Signature.24-34%
Signature.26-15%
Breast #3
fraction
Signature.1-38%
Signature.2-6%
Signature.3-20%
unknown-15%
Signature.13-13%
Signature.26-8%
Breast #5
fraction
Signature.1-53%
unknown-3%
Signature.21-7%
Signature.3-24%
Signature.6-13%
Breast #6
fraction
Signature.1-66%
unknown-3%
Signature.26-9%
Signature.3-7%
Signature.16-16%

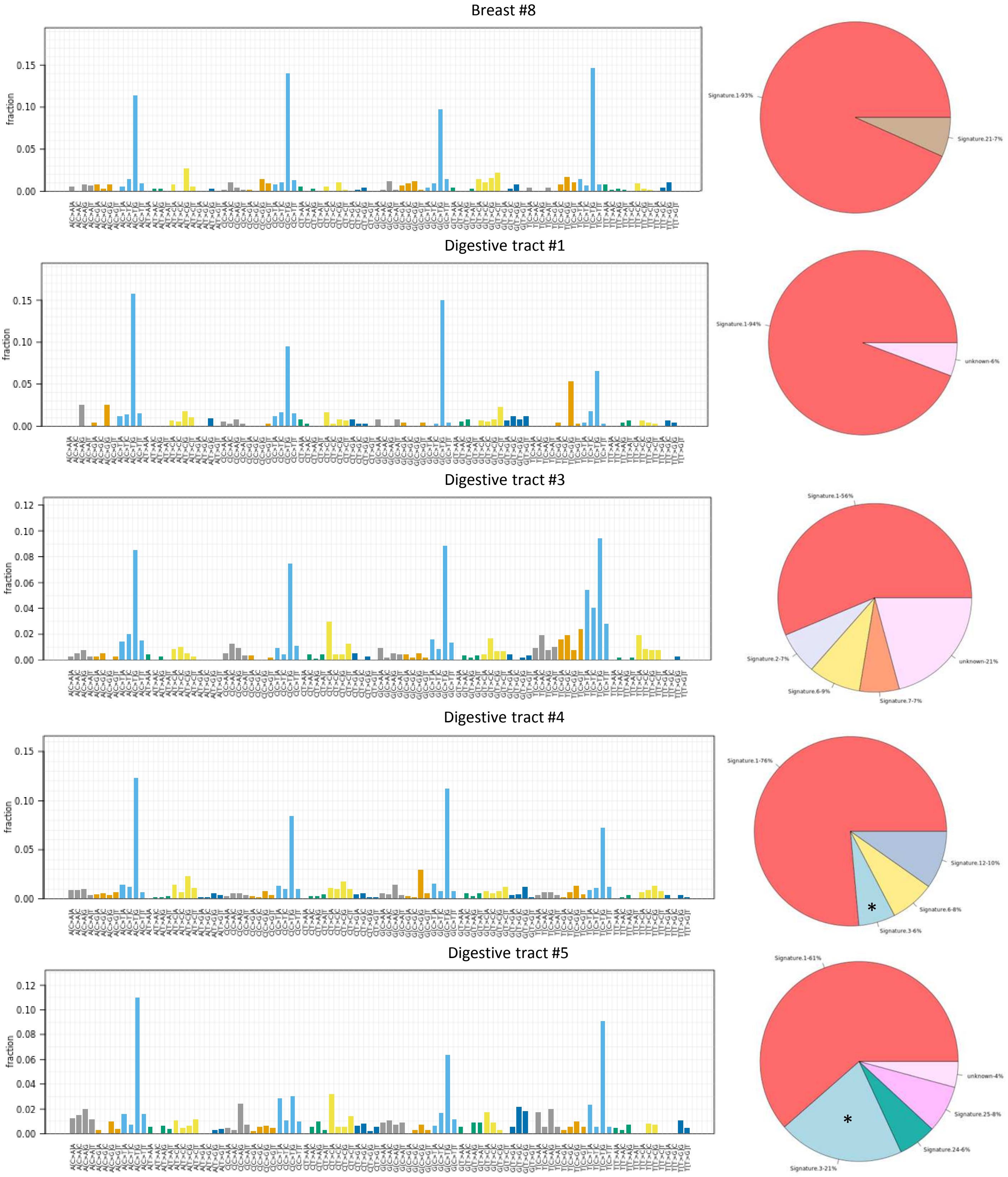
Breast #8
fraction
Signature.1-93%
Signature.21-7%
Digestive tract #1
fraction
Signature.1-94%
unknown-6%
Digestive tract #3
fraction
Signature.1-56%
Signature.2-7%
Signature.6-9%
Signature.7-7%
unknown-21%
Digestive tract #4
fraction
Signature.1-76%
Signature.12-10%
Signature.6-8%
Signature.3-6%
*
Digestive tract #5
fraction
Signature.1-61%
unknown-4%
Signature.25-8%
Signature.24-6%
Signature.3-21%
*

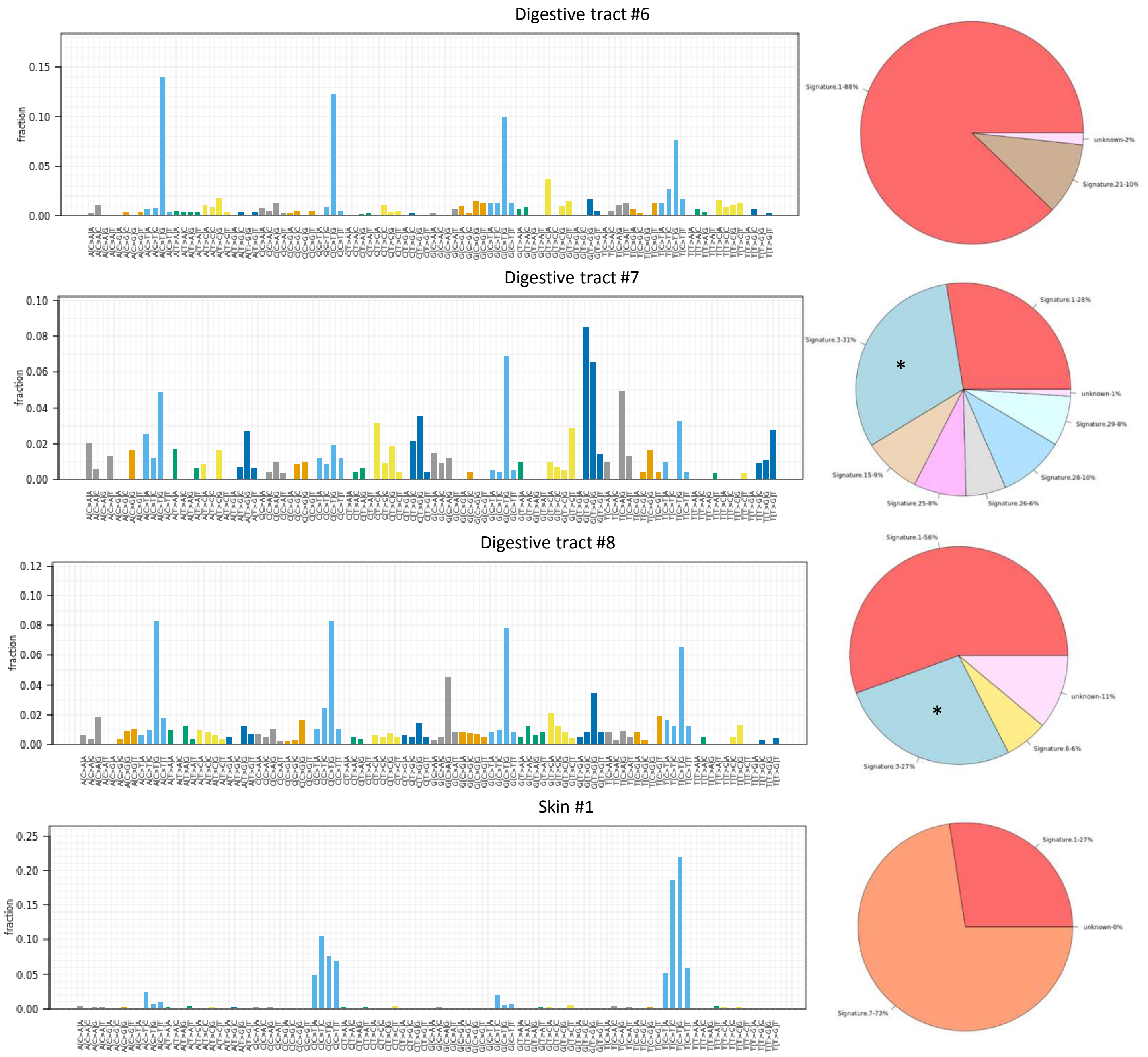
Digestive tract #6
fraction
Signature.1-88%
unknown-2%
Signature.21-10%
Digestive tract #7
fraction
Signature.1-28%
Signature.3-31%
unknown-1%
Signature.29-8%
Signature.28-10%
Signature.26-6%
Signature.25-8%
Signature.15-9%
*
Digestive tract #8
fraction
Signature.1-56%
unknown-11%
Signature.6-6%
Signature.3-27%
*
Skin #1
fraction
Signature.1-27%
unknown-0%
Signature.7-73%

Supplementary Figure S2: Genomic signature profile depending on Alexandrov's work obtained for the 14 patients with both somatic and germline exome analyses. The pie charts indicate the distribution of different signatures bioinformatically detected. Signature 3 is presented in blue and marked with an asterisk.

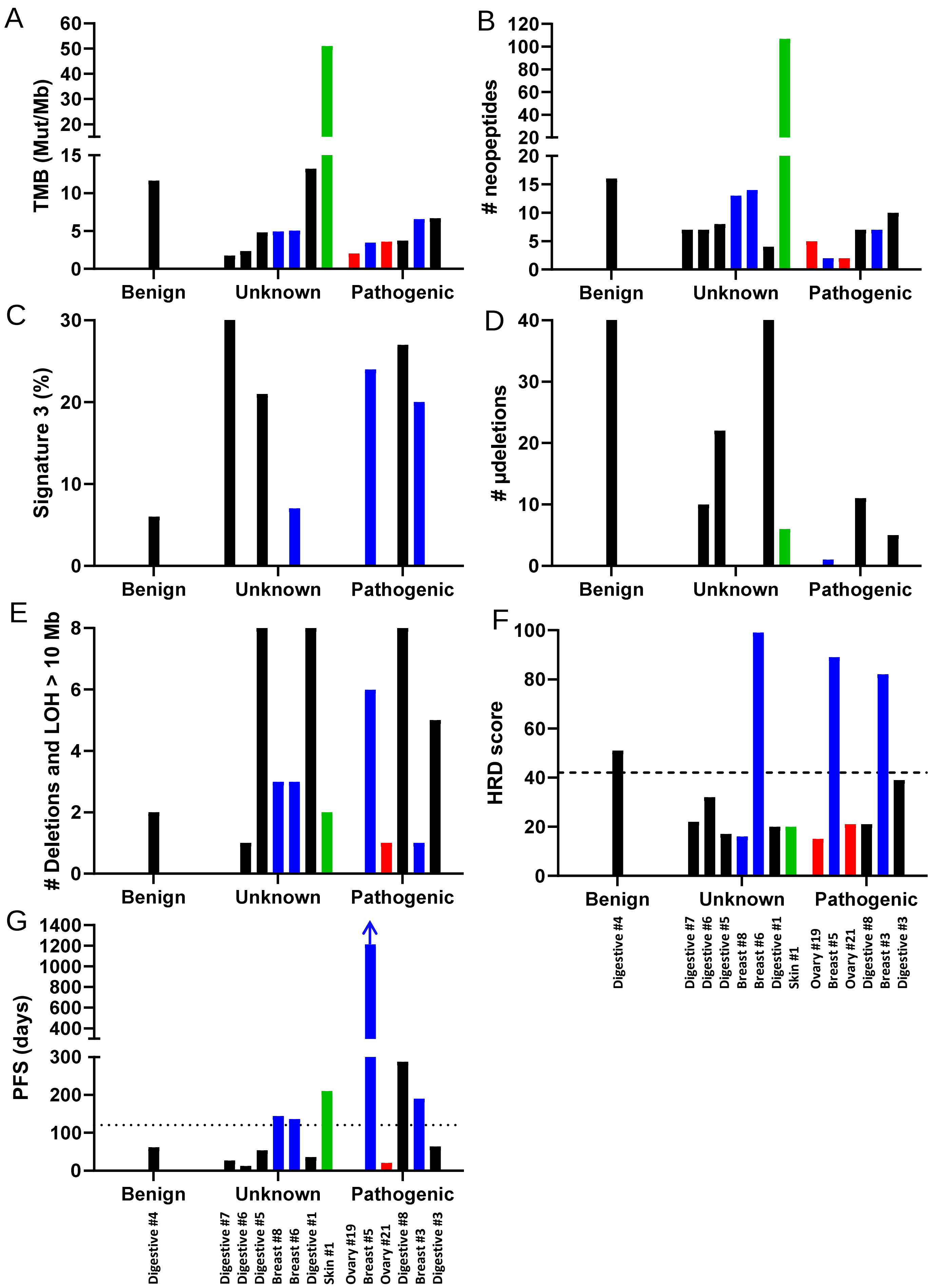

A
TMB (Mut/Mb)
B
neopeptides
C
Signature 3 (%)
D
µdeletions
E
Deletions and LOH > 10 Mb
F
HRD score
G
PFS (days)
Benign
Unknown
Pathogenic
Digestive #4
Digestive #7
Digestive #6
Digestive #5
Breast #8
Breast #6
Digestive #1
Skin #1
Ovary #19
Breast #5
Ovary #21
Digestive #8
Breast #3
Digestive #3

Supplementary Figure S3: Graphical presentation of complex analyses obtained from somatic and germline exomes. A. Tumor Mutation Burden, **B.** number of neopeptides, **C.** Alexandrov's signature 3, **D.** number of small deletions, **E.** number of deletions and loss of heterozygosity (LOH) >10 Mb, **F.** HRD score (dashed line corresponds to the official threshold of 42), and **G.** PFS (dashed line corresponds to 120 days, the arrow indicates that olaparib treatment is still ongoing). Patients are classified depending on the official classification of their variants, and in a group from lower to higher TMB. Patients with a digestive tract cancer are in black, patients with a breast cancer are in blue, patients with an ovary cancer are in red ans patient with a skin cancer is in green.

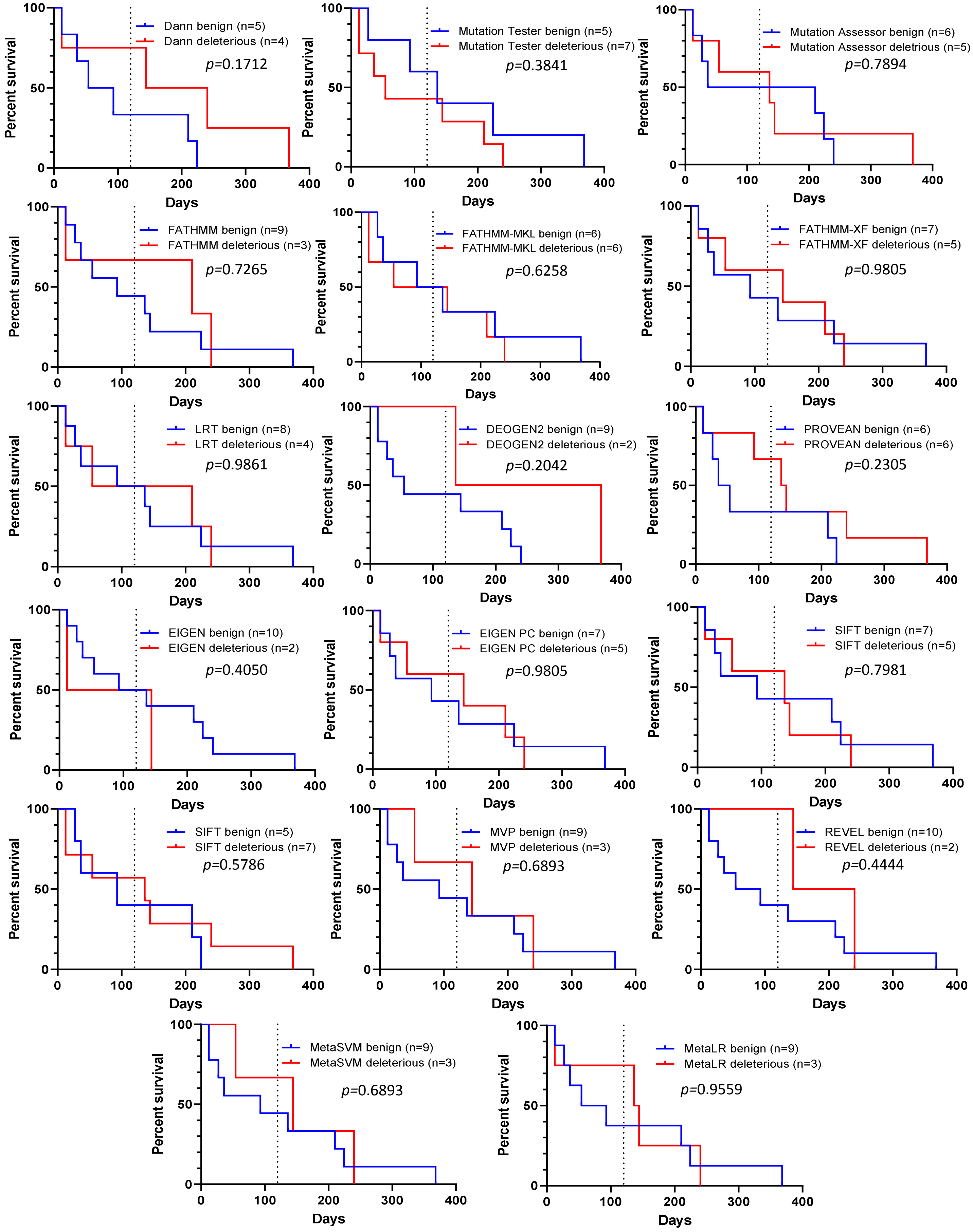
Percent survival
Days
Dann benign (n=5)
Dann deleterious (n=4)
p=0.1712
Mutation Tester benign (n=5)
Mutation Tester deleterious (n=7)
p=0.3841
Mutation Assessor benign (n=6)
Mutation Assessor deletrious (n=5)
p=0.7894
FATHMM benign (n=9)
FATHMM deleterious (n=3)
p=0.7265
FATHMM-MKL benign (n=6)
FATHMM-MKL deleterious (n=6)
p=0.6258
FATHMM-XF benign (n=7)
FATHMM-XF deleterious (n=5)
p=0.9805
LRT benign (n=8)
LRT deleterious (n=4)
p=0.9861
DEOGEN2 benign (n=9)
DEOGEN2 deleterious (n=2)
p=0.2042
PROVEAN benign (n=6)
PROVEAN deleterious (n=6)
p=0.2305
EIGEN benign (n=10)
EIGEN deleterious (n=2)
p=0.4050
EIGEN PC benign (n=7)
EIGEN PC deleterious (n=5)
p=0.9805
SIFT benign (n=7)
SIFT deleterious (n=5)
p=0.7981
SIFT benign (n=5)
SIFT deleterious (n=7)
p=0.5786
MVP benign (n=9)
MVP deleterious (n=3)
p=0.6893
REVEL benign (n=10)
REVEL deleterious (n=2)
p=0.4444
MetaSVM benign (n=9)
MetaSVM deleterious (n=3)
p=0.6893
MetaLR benign (n=9)
MetaLR deleterious (n=3)
p=0.9559
0
50
100
200
300
400

Supplementary Figure S4: PFS under olaparib treatment according to pathogenicity prediction of VUS by 17 *in silico* prediction tools. *In silico* prediction tools are indicated in the legend of each graph. The dashed line corresponds to 120 days.

Supplementary Table S1: Detail of data obtained with the 6 complex analyses from tumor and germline exome sequencing

Cancer type, Patient No.	Gene(s)	Impact	TMB (Mut/Mb)	# Neopeptides	Signature 3 (%)	# µdeletions	# Deletions and LOH > 10 Mb	HRD score	PFS (days)
Ovarian #19	*BRCA2*	Pathogenic	2.03	5	0	0	0	15	1 (allergic reaction)
Ovarian #21	*BRCA2*	Pathogenic	3.58	2	0	0	1	21	288
Breast #3	*BRCA1*	Pathogenic	6.59	7	20	0	1	82	190
Breast #5	*BRCA1*	Pathogenic	3.48	2	24	1	6	89	1212 (still under olaparib)
Breast #6	*BRCA1*	Unknown	5.04	14	7	0	3	99	136
Breast #8	*BRCA2, RAD51D*	Unknown	4.92	13	0	0	3	16	144
Digestive tract #1	*PALB2*	Unknown	13.23	4	0	41	8	20	36
Digestive tract #3	*CHEK2*	Pathogenic	6.7	10	0	5	5	39	64
Digestive tract #4	*BRCA1*	Probably Benign	11.64	16	6	41	2	51	62
Digestive tract #5	*BRCA1*	Unknown	4.82	8	21	22	8	17	54
Digestive tract #6	*BRCA1*	Unknown	2.36	7	0	10	1	32	12
Digestive tract #7	*BRIP1*	Unknown	1.73	7	31	0	0	22	27
Digestive tract #8	*ATM*	Pathogenic	3.75	7	27	11	8	21	20
Skin #1	*PALB2, RAD50, RAD51C*	Unknown	51.04	107	0	6	2	20	210

Supplementary Table S2: Prediction of pathogenicity of VUS using different *in silico* tools

Genes	Nucleotide variation	Protein variation	Dann	Mutation Tester	Mutation Assessor	FATHMM	FATHMM MKL	FATHMM XF	LRT	DEOGEN2	PROVEAN prediction
BRCA1	c.53T>C	p.Met18Thr	Pathogenic	Disease causing	Neutral	Damaging	Damaging	Damaging	Deleterious	Tolerated	Damaging
PALB2	c.656A>G	p.Asp219Gly	Neutral	Polymorphism	Neutral	Tolerated	Neutral	Neutral	Neutral	Tolerated	Neutral
BRCA1	c.2744C>T	p.Ser915Phe	Pathogenic	Polymorphism	Medium	Tolerated	Neutral	Neutral	Neutral	Damaging	Damaging
BRCA2	c.1690T>C	p.Met990Lys	Neutral	Polymorphism	Unknown	Tolerated	Neutral	Neutral	Neutral	Unknown	Damaging
BRCA1	c.2783G>T	p.Gly928Val	Unknown	Polymorphism	Medium	Tolerated	Neutral	Neutral	Neutral	Damaging	Damaging
BRCA2	c.4860A>T	p.Leu1620Phe	Neutral	Polymorphism	Unknown	Tolerated	Neutral	Neutral	Neutral	Unknown	Damaging
RAD51D	c.328G>A	p.Asp110Asn	Pathogenic	Disease causing	Medium	Tolerated	Damaging	Damaging	Neutral	Tolerated	Damaging
PALB2	c.2719G>A	p.Glu907Lys	Neutral	Disease causing	Low	Tolerated	Neutral	Neutral	Neutral	Tolerated	Neutral
BRCA1	c.2521C>T	p.Arg841Trp	Neutral	Polymorphism	Low	Tolerated	Neutral	Neutral	Neutral	Tolerated	Damaging
UIMC1	c.1690T>C	p.Tyr564His	Pathogenic	Disease causing	Medium	Tolerated	Damaging	Damaging	Deleterious	Tolerated	Damaging
BRCA1	c.5128A>C	p.Met1710Leu	Neutral	Disease causing	Medium	Tolerated	Damaging	Damaging	Deleterious	Tolerated	Neutral
BRCA1	c.5295A>C	p.Glu1786Asp	Unknown	Disease causing	Low	Damaging	Damaging	Neutral	Neutral	Tolerated	Neutral
BRIP1	c.3G>A	p.Met1?	Neutral*	Neutral*	Neutral*	Neutral*	Neutral*	Neutral*	Neutral*	Neutral*	Neutral*
PALB2	c.2431C>T	p.Pro811Ser	Neutral	Polymorphism	Low	Tolerated	Damaging	Neutral	Neutral	Tolerated	Neutral
RAD50	c.3041A>G	p.Gln1014Arg	Neutral	Disease causing	Low	Damaging	Damaging	Damaging	Deleterious	Tolerated	Neutral
RAD51C	c.584C>T	p.Ala195Val	Neutral	Disease causing	Low	Tolerated	Damaging	Neutral	Deleterious	Tolerated	Neutral

*New in frame initiation codon, 3 codons later.

Genes	Nucleotide variation	Protein variation	EIGEN	EIGEN PC	SIFT	SIFT4G	MVP	REVEL	Primate AI	MetaSVM	MetaLR
BRCA1	c.53T>C	p.Met18Thr	Benign	Pathogenic	Damaging	Damaging	Pathogenic	Pathogenic	Tolerated	Damaging	Damaging
PALB2	c.656A>G	p.Asp219Gly	Benign	Benign	Tolerated	Tolerated	Benign	Benign	Tolerated	Tolerated	Tolerated
BRCA1	c.2744C>T	p.Ser915Phe	Benign	Benign	Tolerated	Damaging	Benign	Benign	Tolerated	Tolerated	Tolerated
BRCA2	c.1690T>C	p.Met990Lys	Benign	Benign	Tolerated	Tolerated	Benign	Benign	Tolerated	Tolerated	Tolerated
BRCA1	c.2783G>T	p.Gly928Val	Benign	Benign	Damaging	Damaging	Benign	Benign	Tolerated	Tolerated	Damaging
BRCA2<br>*RAD51D*	c.4860A>T<br>c.328G>A	p.Leu1620Phe<br>p.Asp110Asn	Benign<br>Pathogenic	Benign<br>Pathogenic	Damaging<br>Damaging	Tolerated<br>Damaging	Benign<br>Pathogenic	Benign<br>Pathogenic	Tolerated<br>Tolerated	Tolerated<br>Damaging	Tolerated<br>Damaging
PALB2	c.2719G>A	p.Glu907Lys	Benign	Benign	Tolerated	Tolerated	Benign	Benign	Tolerated	Tolerated	Tolerated
BRCA1<br>*UIMC1*	c.2521C>T<br>c.1690T>C	p.Arg841Trp<br>p.Tyr564His	Benign<br>Pathogenic	Benign<br>Pathogenic	Damaging<br>Damaging	Damaging<br>Damaging	Benign<br>Benign	Benign<br>Benign	Tolerated<br>Tolerated	Tolerated<br>Tolerated	Tolerated<br>Tolerated
BRCA1	c.5128A>C	p.Met1710Leu	Benign	Pathogenic	Damaging	Damaging	Pathogenic	Benign	Tolerated	Damaging	Tolerated
BRCA1	c.5295A>C	p.Glu1786Asp	Benign	Benign	Tolerated	Damaging	Benign	Benign	Tolerated	Tolerated	Damaging
BRIP1	c.3G>A	p.Met1?	Neutral*	Neutral*	Neutral*	Neutral*	Neutral*	Neutral*	Neutral*	Neutral*	Neutral*
PALB2<br>*RAD50*<br>*RAD51C*	c.2431C>T<br>c.3041A>G<br>c.584C>T	p.Pro811Ser<br>p.Gln1014Arg<br>p.Ala195Val	Benign<br>Benign<br>Benign	Benign<br>Benign<br>Pathogenic	Tolerated<br>Unknown<br>Tolerated	Tolerated<br>Tolerated<br>Tolerated	Benign<br>Benign<br>Benign	Benign<br>Benign<br>Benign	Tolerated<br>Tolerated<br>Tolerated	Tolerated<br>Tolerated<br>Tolerated	Tolerated<br>Tolerated<br>Tolerated

*New in frame initiation codon, 3 codons later.
